# Eggs as a Nutrient-Rich Food with Potential Relevance to Sleep Metabolic Health, and Well-Being During the Menopausal Transition: A Narrative Review

**DOI:** 10.3390/nu17243837

**Published:** 2025-12-08

**Authors:** Lilia Convit, Christa-Marie Nicola, Charles S. Urwin, Spencer S. H. Roberts, Sze-Yen Tan, Samantha M. Hoffmann, Dominique Condo, Robin M. Daly, D. Lee Hamilton, Rhiannon M. J. Snipe

**Affiliations:** 1 Institute for Physical Activity and Nutrition, School of Exercise and Nutrition Sciences, Faculty of Health, Deakin University, Geelong, VIC 3216, Australia; lilia.convitcordova@deakin.edu.au (L.C.); c.urwin@deakin.edu.au (C.S.U.); s.roberts@deakin.edu.au (S.S.H.R.); szeyen.tan@deakin.edu.au (S.-Y.T.); s.hoffmann@deakin.edu.au (S.M.H.); dominique.condo@deakin.edu.au (D.C.); robin.daly@deakin.edu.au (R.M.D.); lee.hamilton@deakin.edu.au (D.L.H.); 2 School of Exercise and Nutrition Sciences, Faculty of Health, Deakin University, Geelong, VIC 3216, Australia

**Keywords:** perimenopause, dietary protein, choline, antioxidants, sleep quality, body composition

## Abstract

Perimenopause and the menopausal transition are characterised by hormonal fluctuations that disrupt thermoregulation, metabolism, and sleep, contributing to adverse changes in body composition and increased cardiometabolic risk. Despite these challenges, food-based strategies to support sleep, appetite regulation, and metabolic health remain underexplored. This narrative review synthesised current evidence on the nutritional factors influencing these outcomes, with emphasis on the potential role of eggs as a nutrient-dense, accessible dietary option for midlife women. Literature searches identified studies examining hormonal mechanisms and the effects of nutrients abundant in eggs, including high-quality protein, choline, tryptophan, melatonin, vitamin D, and antioxidants. Evidence suggests that adequate protein and choline intake may enhance sleep duration, satiety, and preserve lean mass, while vitamin D and antioxidant compounds may support muscle function and mitigate oxidative stress associated with hormonal decline. Collectively, eggs represent a practical whole-food source of nutrients that may play a role in supporting sleep, appetite regulation, and body-composition maintenance during the menopausal transition; however, further high-quality intervention studies are needed to confirm these effects.

## 1. Introduction

Across the lifespan, women undergo distinct reproductive stages, largely influenced by fluctuations in reproductive hormones [1]. These transitions begin with puberty, marking the onset of reproductive capacity, and culminate in menopause, defined as the cessation of menstruation for more than one year, which typically occurs at an average age of around 51 years [1]. Before menopause, there is a transitional phase known as perimenopause, marked by a gradual decline in ovarian function and fluctuating levels of hormones such as oestrogen, progesterone, and testosterone [2], unless menopause is medically or surgically induced from a premenopausal state [3]. Perimenopause typically begins in the early to mid-forties and lasts approximately 4–10 years [1]. During early perimenopause, menstrual cycles may become irregular, with differences of 7 days or more between consecutive cycles, whereas late perimenopause is characterised by longer gaps between periods (typically ≥60 days) [2,4,5]. These hormonal changes are associated with a wide range of symptoms, including sleep disturbances, irregular menses, hot flushes, mood swings, depression and fatigue, which can collectively reduce quality of life [6]. It is estimated that 80–90% of women experience one or more of these or similar symptoms during perimenopause [4].

Despite its profound impact on quality of life, perimenopause remains under-researched and poorly recognised in both clinical and research contexts, particularly due to the challenges in its identification and definition [7,8]. This oversight contributes to a broader gap in women’s health research compared to men’s, especially in relation to sleep, which is commonly disrupted during this life stage [9]. The physiological and biological differences between women and men already predispose women to a 40% higher risk of developing insomnia (i.e., difficulty initiating or maintaining sleep) [9], and this risk is further exacerbated by hormonal fluctuations across the perimenopausal transition. Approximately 40–60% of women report insomnia during perimenopause, with over half experiencing reduced sleep duration (<7 h per night) or poor sleep quality (i.e., non-restorative or fragmented sleep despite adequate opportunity) [4,7]. Other forms of sleep disturbance, including increased sleep latency, frequent night awakenings, and reduced sleep efficiency, are also commonly reported in this life phase [7,10]. Poor sleep has been associated with increased anxiety, metabolic disturbances [11], and greater hunger and appetite [12], which may further exacerbate perimenopausal symptoms and contribute to increased disease risks associated with weight gain.

Given the high prevalence of these multifaceted sleep disturbances (i.e., non-clinical sleep complaints such as poor sleep quality, short duration, or difficulty maintaining sleep) in perimenopausal women, there is increasing interest in identifying accessible, evidence-based strategies to improve sleep quality [13]. In this context, adopting a whole-food approach that incorporates strategic nutrient timing may provide a promising avenue for managing perimenopausal symptoms. With respect to sleep, the timing and composition of evening meals that include key sleep-supporting nutrients such as tryptophan and melatonin may be particularly relevant.

**Figure 1:**
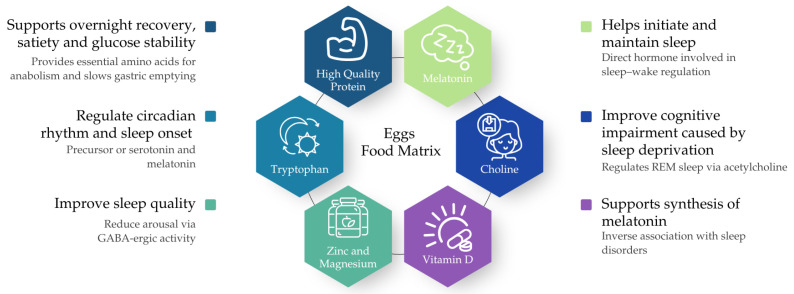
Sleep-relevant nutrients within the egg food matrix [16,17,18,19,20,21,22,23,24,25,26,27,28,29].

At this life stage, hormonal fluctuations associated with declining oestrogen can disrupt sleep and metabolism, highlighting the importance of adequate dietary protein to support sleep quality and overnight muscle repair [14]. However, in Australia and other Organisation for Economic Co-operation and Development (OECD) countries, women in this age group tend to consume most of their protein earlier in the day, with total intakes averaging ~80–90 g·day^−1^ (~1.0–1.2 g·kg^−1^ day^−1^), which meets but may not optimise requirements for muscle maintenance and recovery [15]. Given that the overnight period, typically lasting six to eight hours, represents the longest interval most people go without consuming food, the pre-sleep period has garnered increased attention not necessarily to increase total protein intake, but to improve its distribution and timing to support overnight recovery processes and possibly sleep regulation [16]. Chicken eggs are a rich source of high-quality, anabolic protein [16] in addition to other nutrients of interest, including tryptophan, melatonin, vitamin D, and choline, each of which has been individually associated with improved sleep outcomes [17,18,19]. Although these nutrients are present in a range of other protein-rich foods, eggs are unique in providing several sleep-relevant nutrients together within a single whole food, making them a practical model for considering potential food-matrix effects (Figure 1). These compounds may influence sleep through several distinct physiological pathways. For example, tryptophan is a precursor to serotonin and melatonin, which help regulate circadian rhythms and promote sleep onset [20,21,22]. Choline plays a role in the synthesis of acetylcholine, a neurotransmitter involved in the initiation and maintenance of Rapid Eye Movement (REM) sleep [23]. Vitamin D may contribute to melatonin regulation and has been inversely associated with sleep disorders in observational studies [24,25,26,27,28]. Zinc and magnesium, also present in chicken eggs, are thought to enhance sleep quality by modulating gamma-aminobutyric (GABA)-ergic activity and reducing arousal [29]. However, the quantities of these nutrients naturally present in eggs have not been compared with the doses shown to influence sleep outcomes in controlled studies, so these mechanistic pathways should be interpreted cautiously.

As illustrated in Figure 1, these pathways remain theoretical and reflect nutrient-based hypotheses rather than established clinical effects of egg consumption in perimenopausal women. The presence of multiple sleep-influencing compounds in chicken eggs may provide synergistic benefits through the so-called “food-matrix” effect, which may not be achieved to the same extent with foods supplying only some of these nutrients. Such potential synergistic effects, particularly when eggs are consumed in the evening, given the closer temporal proximity to sleep onset, remain speculative. Therefore, pre-sleep or evening consumption of eggs may serve as a tool to improve overall high-quality protein intake and could plausibly support sleep, but this has not yet been tested in well-designed intervention.

More broadly, the current evidence in this area is limited, with very few randomised controlled trials examining whole-food interventions, including eggs, on sleep outcomes. Most available studies are observational or evaluate isolated nutrients, which restricts causal interpretation. Inconsistent findings likely reflect differences in study design, sleep outcome definitions, dietary assessment methods, and participant characteristics such as menopausal stage and baseline sleep quality. Importantly, the optimal dosage and timing of sleep-relevant nutrients, or for whole foods such as eggs, remain unknown, representing key gaps for future research.

This narrative review explores the potential role of chicken eggs (hereafter referred to simply as “eggs”) in supporting sleep quality among perimenopausal women. Where research directly investigating eggs in this context is limited, relevant evidence from broader populations is examined, alongside studies evaluating individual nutrients found in eggs. Additionally, the review considers how egg consumption may influence other sleep-related perimenopausal concerns, such as mood disturbances, vasomotor symptoms (e.g., hot flushes and night sweats), increased appetite, and adverse changes in body composition, including loss of lean mass and increased fat mass, ultimately supporting healthier ageing in midlife women. An initial search in PubMed, Scopus, Medline Complete and Embase using predefined population, intervention, and outcome terms returned more than 10,000 records, with only a small number of titles appearing potentially relevant after preliminary screening. The scarcity of eligible studies and the heterogeneity of designs and outcomes precluded a systematic review, and a narrative approach was therefore adopted. Full search terms and criteria are provided in Table S1.

## 2. Hormonal Changes, Sleep Disruption, and Metabolic Health in Midlife Women

Sleep disorders are highly prevalent among midlife women, affecting 16–47% of those in perimenopause and up to 60% of those in postmenopause [4,7]. These sleep disturbances encompass insomnia, poor sleep quality, increased sleep onset latency [30], fragmented sleep, frequent night awakenings [31], and short sleep duration (<7 h per night) [7,10]. These sleep disturbances may be driven by a combination of disrupted circadian rhythms, elevated cortisol levels, psychological stress, irregular routines (e.g., shift work), and poor sleep hygiene [7,30] and are consistently associated with impaired work productivity and quality of life [32]. However, the underlying causes of these sleep problems during perimenopause are thought to be largely hormonal [33]. Reduced and fluctuating concentrations of the sex steroid hormones oestrogen and progesterone during perimenopause influence sleep regulation, thermoregulation, mood, and metabolism [8,34,35]. Oestrogen contributes to serotonin production and helps lower core body temperature [36] while progesterone exerts a mild sedative effect and influences thermoregulatory pathways in the hypothalamus [36,37]. When oestrogen levels decline, thermoregulatory instability can occur, triggering hot flushes and night sweats that interrupt sleep, particularly by prolonging sleep onset latency and causing frequent nocturnal awakenings [34,38]. In addition, mood changes, musculoskeletal discomfort, and genitourinary symptoms commonly experienced during perimenopause (Table 1) may further impair sleep continuity and overall quality. In parallel, inherent sex-based physiological differences and psychological stress, and lifestyle factors amplify women’s susceptibility to insomnia, about 40% higher than in men [39], particularly during perimenopause when hormonal variability is greatest [8,40,41].

**Figure 2:**
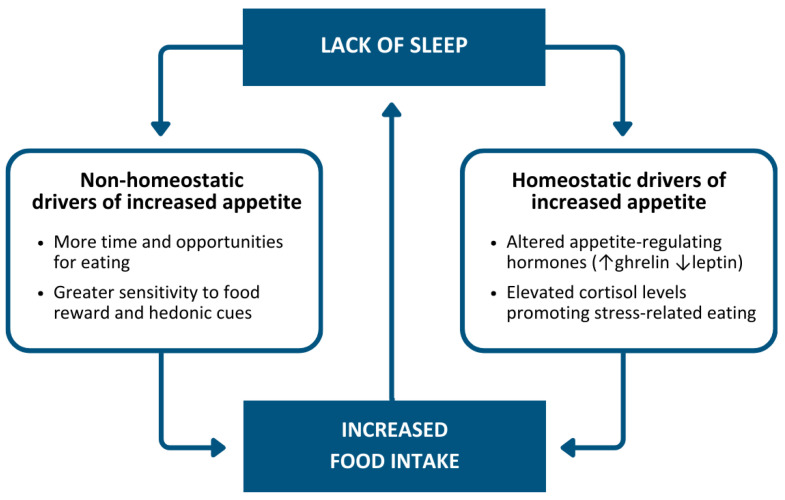
Relationship between lack of sleep and increased food intake. Adapted from: [57,58].

Chronic sleep deprivation, defined as persistent difficulty sleeping for more than three months [45], has wide-ranging physiological and psychological consequences. It impairs emotional regulation, heightens stress reactivity, and increases anxiety, accompanied by elevated cortisol and altered neurotransmitter activity involved in mood regulation (e.g., serotonin and dopamine) [46,47]. In perimenopausal women, inadequate or poor-quality sleep further disrupts appetite-regulating hormones, raising ghrelin (hunger hormone) and cortisol while lowering leptin (satiety hormone), promoting increased appetite and preferences for energy-dense foods (Figure 2) [48,49]. Over time, this contributes to adverse body composition shifts, including central (visceral) fat accumulation, insulin resistance, and systemic inflammation, thereby increasing cardiometabolic risk [48,50,51]. Insufficient deep sleep also interferes with growth hormone release, which is critical for tissue repair, muscle growth, and metabolic regulation [52]. Reduced growth hormone secretion impairs recovery from exercise, diminishes muscle preservation and accelerates age-related losses of lean mass and bone density [48,50,51,53,54]. Combined with age-related losses in muscle and bone mass, which increase the risk of sarcopenia and osteopenia/osteoporosis, poor sleep may amplify physical frailty and metabolic dysfunction in perimenopausal women [50,51]. Moreover, disrupted sleep contributes to fatigue, cognitive difficulties (brain fog), mood swings, and depression, intensifying the overall symptom burden of the menopausal transition [7,40,55,56].

## 3. Dietary Strategies to Support Sleep and Well-Being

Despite the high prevalence of sleep disturbances and other symptoms during perimenopause, few studies have comprehensively explored dietary strategies as non-pharmacological interventions to mitigate these side effects [7]. Emerging evidence suggests that targeted dietary components may play a beneficial role in sleep regulation during the menopausal transition, particularly through their influence on neurotransmitter pathways and circadian rhythms [21,57,59]. Chrono-nutrition, which examines the timing, frequency, and distribution of meals aligned with circadian rhythms, is particularly relevant for perimenopausal women, whose circadian regulation, and consequently sleep patterns, may already be disrupted by hormonal fluctuations [60,61].

Within the broader chrono-nutritional framework of diet and meal timing, protein has attracted attention because it provides essential amino acids such as tryptophan, a precursor to serotonin and melatonin, both of which are critical for sleep–wake regulation [21,22]. Evening consumption of protein-rich foods may be especially beneficial, supporting endogenous melatonin production and providing additional sleep-related nutrients such as vitamin D and choline [25,26,27,28]. In addition to its direct role in neurotransmitter pathways, a higher proportion of dietary protein within an isoenergetic diet has also been associated with the preservation or attenuation of declines in lean mass and modest reductions in fat mass, and enhanced appetite control [62,63,64], which is particularly important during perimenopause, when factors associated with hormonal fluctuations (Table 1), including vasomotor symptoms, fatigue, and reduced physical activity, may indirectly contribute to muscle loss and increased the risk of sarcopenia [44,65,66]. The reduction in fat mass may indirectly support sleep quality by reducing central (visceral) adiposity and improving metabolic health, both of which are commonly disrupted during perimenopause [49]. Excess adiposity, particularly in the abdominal region, is associated with poorer sleep quality and an increased risk of sleep-disordered breathing such as obstructive sleep apnoea [67], conditions that often emerge or worsen during midlife and contribute to the broader cardiometabolic profile that influences sleep and well-being. Weight reduction has been shown to improve sleep outcomes primarily through decreases in airway obstruction, inflammation, and metabolic strain [68]. In contrast, evidence that greater lean mass directly enhances sleep quality is limited, and improvements are more plausibly mediated by reductions in fat mass rather than increases in muscle tissue. Conversely, inadequate sleep can impair skeletal-muscle anabolism; for example, a recent study demonstrated that a single night of total sleep deprivation reduced post-prandial muscle-protein synthesis by ~18% and altered anabolic hormone profiles [49], highlighting the bidirectional relationship between sleep and body composition regulation.

A growing body of research has explored the relationship between nutrition and sleep, yet evidence specific to perimenopausal women remains limited. A 2023 systematic review identified 59 studies examining nutritional interventions and sleep outcomes, but only three focused specifically on perimenopausal women; the remainder investigated menopausal (*n* = 18), postmenopausal (*n* = 24) or mixed menopausal-status cohorts (*n* = 14) [69]. The review reported potential benefits of isoflavones (e.g., soy-based foods) for improving subjective sleep outcomes, as well as black cohosh for reducing wake time after sleep onset in postmenopausal women [69]. Findings for other interventions, including resveratrol and omega-3 fatty acids supplementation, tested across peri-to postmenopausal groups, were inconsistent [43,69]. Overall, these findings underscore the need for rigorous, well-designed studies in perimenopausal women.

Complementary observational data suggest that habitual nutrient intake may also influence sleep. For instance, an analysis of 1116 women aged 40–59 years found that lower intake of protein, carbohydrates, thiamine, folate, choline, phosphorus, sodium, potassium, and selenium was associated with shorter sleep duration [70]. However, menopausal status in that study was inferred by age rather than symptom profile, and sleep duration was self-reported, introducing potential personal bias [70].

Although hormonal changes during perimenopause can influence energy balance, appetite and nutrient metabolism, research into actual dietary habits in this population remains limited. A 5-year longitudinal study using self-reported dietary intake via food diaries reported that perimenopausal women (*n* = 94, mean age 49.9 ± 1.9 years) reduced their intake of energy, protein, fat and fibre; nutrients essential for regulating mood, satiety, and sleep through their roles in neurotransmitter synthesis and blood glucose stability, despite experiencing increased appetite [57,59]. Whole-food-based strategies (e.g., eggs) that provide a complete amino acid profile and key micronutrients, such as choline, magnesium, and vitamin D, may therefore represent accessible and sustainable approaches to support or optimise sleep quality during perimenopause [59,71].

## 4. The Nutritional Value of Eggs in Midlife Health

Building on evidence that targeted nutrients can influence sleep and overall well-being, eggs represent a practical, nutrient-dense dietary strategy for perimenopausal women experiencing sleep disturbances. They are naturally rich in high-quality protein, tryptophan, melatonin, vitamin D, and choline, nutrients that are fundamental to sleep regulation, neurotransmitter production, mood stability, and metabolic health [26,72]. Each chicken egg provides approximately 6.3 g of complete protein (Table 2), which becomes increasingly important in attenuating the potential muscle loss during the perimenopausal transition [1,73]. Importantly, eggs deliver these key nutrients together in a single whole-food matrix, promoting nutrient synergy, bioavailability, and dietary adherence compared with isolated supplementation. Eggs are also one of the few natural food sources of vitamin D, a nutrient that plays a key role in not only bone health but also mood regulation and circadian rhythm synchronisation [28,38,44,73,74]; whereas other natural sources, such as oily fish and liver, may be less accessible or less commonly consumed. In addition, eggs contain choline and tryptophan, which serve as precursors to neurotransmitters such as acetylcholine, serotonin, and melatonin, which are involved in cognitive function, mood, and sleep onset [75,76]. Together, these properties position eggs as an accessible, whole-food option to support and optimise sleep quality during perimenopause.

While this review focuses primarily on chicken eggs, which account for approximately 93% of the global egg production and consumption [77], other types, such as quail and duck eggs, also have distinct nutritional profiles (Table 2). Compared with chicken eggs, quail eggs contain slightly higher amounts of protein and certain micronutrients (iron, copper, magnesium, phosphorus), while duck eggs are more energy-dense and contain greater amounts of fat, cholesterol, and fat-soluble vitamins (A, D, E, K) [78,79]. It is also important to note that nutrient composition can vary within chicken eggs depending on production systems (e.g., free-range, barn-laid) and enrichment practices (e.g., omega-3 or vitamin D). Nonetheless, these compositional differences are relatively small in the context of a mixed diet and are unlikely to translate into meaningful differences in health outcomes. Given that chicken eggs are the most widely available, affordable, and well-studied type globally, they represent the most practical model for evaluating potential food-based strategies to support sleep and metabolic health in perimenopausal women. To date, no studies have directly compared the effects of different egg types on sleep or well-being, making this an area worthy of future investigation.

**Table 2:** Nutritional profile of chicken, quail, and duck eggs.

	Chicken Egg	Quail Egg	Duck Egg
Component			
Moisture (%)	74.8	72.9	70.8
Protein (%)	11.9	12.9	13.0
Fats (%)	10.6	11.4	14.4
Ash (%)	0.9	1.0	1.1
Carbohydrates (%)	1.6	1.6	1.3
Energy (Kcal·100 g^−1^)	149.9	161.1	185.0
Cholesterol (mg·g^−1^)	12.4	12.2	13.0
Omega-3 (mg·100 g^−1^)	50.0	80.0	150.0
Mineral (mg·100 g^−1^)			
Calcium	35.4	31.4	64.0
Iron	2.8	3.0	3.8
Copper	1.9	2.2	0.2
Zinc	3.5	3.1	1.6
Magnesium	17.3	19.8	16.0
Sodium	31.8	25.7	146.0
Potassium	23.8	20.8	222.0
Phosphorus	236.3	302.5	220.0
Vitamins			
A (µg·100 g^−1^)	140.0	156.0	192.0
D (µg·100 g^−1^)	2.0	1.4	2.0
E (mg·100 g^−1^)	1.3	1.1	2.0
K (µg·100 g^−1^)	0.4	0.3	0.6

Values represent standard eggs. Nutrient composition varies with feed composition, pasture access, and commercial enrichment. Vitamin K is present in very small amounts across all egg types, making practical dietary contribution minimal. NR: not reported. Adapted from: [78,79,80].

From a practical standpoint, eggs are accessible, affordable, widely accepted, and versatile, making them a convenient addition to the diet across cultural and lifestyle contexts. Chicken eggs represent almost all egg consumption in Australia and globally [81], reflecting their affordability and year-round availability compared with duck or quail eggs. National data indicate that egg consumption in Australia has increased by around 35% over the past decade [81], supporting their position as a familiar, low-cost source of high-quality protein and micronutrients. These characteristics make chicken eggs a realistic vehicle for implementing food-based strategies targeting sleep and metabolic health in perimenopausal women. While breakfast remains the most common eating occasion, the data highlight that eggs are also frequently consumed later in the day [82], which is relevant given that evening egg intake may enhance their potential sleep-promoting effects by optimising the metabolism of tryptophan and melatonin to support sleep onset and duration [64,75,83]. Following ingestion, plasma tryptophan peaks within approximately 1–2 h and can be converted to serotonin and subsequently to melatonin, which reaches maximal circulating concentrations within about 20–60 min before being rapidly metabolised, with a biological half-life of ~30–60 min [84,85,86]. These kinetics support the rationale that consuming melatonin- and tryptophan-containing foods, such as eggs, in the evening may better align with nocturnal melatonin synthesis and sleep duration and quality [87]. As such, incorporating moderate egg evening consumption (~4–5 times per week) [88] may therefore offer a simple strategy for potentially improving sleep during perimenopause.

While there is limited research directly examining egg intake and sleep outcomes in perimenopausal women, several observational studies in mixed-sex and age populations provide preliminary insights [89,90]. Supportive evidence comes from large cross-sectional cohorts, where dietary patterns rich in nutrient-dense foods, among them eggs, were associated with reduced insomnia risk [89]. In a Chinese cohort of 481,000 women (mean age 51.5 years), a modern dietary pattern characterised by higher intakes of eggs along with meat, poultry, fish, fruit, and dairy was linked to fewer insomnia symptoms, including difficulty initiating or maintaining sleep and early morning awakenings [89]. However, because these studies examined whole dietary patterns, it is not possible to disentangle the specific contribution of eggs from other foods known to influence sleep, such as oily fish or dairy. Similarly, in a study of 215 Japanese women aged 42–70 years, adherence to a healthy dietary pattern including frequent egg intake was associated with fewer instances of difficulty falling asleep [90]. A small pilot study in 24 U.S. female soccer players (Division IA) also reported that higher egg consumption (≥3.5 eggs per week) was linked to better subjective sleep quality via the Pittsburgh Sleep Quality Index (PSQI), although the younger, athletic population may limit generalisability to perimenopausal women [91]. In contrast, a study of 996 Chinese women over 45 years found that greater egg intake was associated with poorer sleep quality as measured by the PSQI, yet, as with most observational designs, residual confounding from overall diet quality and lifestyle factors cannot be excluded [92]. Specifically, women with better overall diet quality, characterised by greater food variety, higher fruit and seafood intake, and lower egg consumption, had a significantly lower risk of poor sleep quality, even after adjusting for age, stress, smoking, hypertension and body mass index. These findings may differ from other studies due to cultural variations in dietary patterns, primary protein sources, and meal timing, which limit generalisability to Western populations. This study also found that higher egg intake was correlated with elevated serum cholesterol levels (r = 0.283, *p* < 0.001); however, this increase did not translate into poorer sleep quality, unlike prior reports of cholesterol-sleep associations observed mainly in men [92,93]. Given that eggs are high in cholesterol but relatively low in saturated fat and are consumed within diverse dietary contexts that can alter overall nutrient balance, differences in accompanying foods and dietary patterns may partly explain the inconsistent findings across studies [94,95,96,97].

Taken together, these findings highlight emerging but inconclusive evidence linking egg intake and sleep quality and duration. While eggs consistently appear within dietary patterns associated with better sleep, study designs relying on self-reported dietary intake and sleep outcomes, as well as broad age ranges and cultural differences, limit their applicability to perimenopausal women. Controlled trials are therefore needed to clarify the timing, quantity, and physiological effects of egg-based interventions in this group.

## 5. Key Egg-Derived Nutrients and Their Roles in Sleep, Appetite, and Body Composition

Building on the preceding section on eggs as a whole food, this section examines the specific effects of individual egg-derived nutrients on mechanisms related to sleep, appetite, and body composition.

### 5.1. Protein and Choline

Each large egg provides ~6.3 g of protein and ~150 mg of choline (~35% of the daily requirement for women; Table 2), making it a convenient whole-food source of nutrients linked to neurotransmitter function and sleep regulation [26,73,98]. Sleep quality is a critical determinant of overall health and longevity, and emerging evidence suggests that dietary protein from dairy, eggs, meat and soy may help regulate sleep duration, latency, and efficiency [73]. A 2019 systematic review of 19 studies in adults aged ≥19 years reported that higher protein intake was generally associated with better sleep outcomes; notably, 12 studies included women aged over 40 years, supporting relevance to perimenopausal populations [14]. Good sleepers (≥7 h/night) consumed a greater proportion of energy from protein (16–34%) than poor sleepers (10–20%), a relationship thought to be mediated by tryptophan’s role in serotonin and melatonin synthesis [14]. However, causality remains unconfirmed, and well-controlled randomised trials are needed to validate these associations. More recent data from a 2024 cross-sectional study of 4825 adults (81.6% women; mean age 36.7 years) further support this link [99]. Using combined data from two mobile applications (Pokémon Sleep, a gamified sleep tracking tool, and Asken, a diet/lifestyle tracker), higher protein intake (21–24% of daily energy, quartiles 3 and 4) was associated with ~10 min longer sleep duration compared with the lowest protein quartile (~15–17% of energy from protein) [99]. Although statistically significant, this modest difference is unlikely to be clinically meaningful, as changes of ≥20–30 min in total sleep time are typically considered relevant for improvements in sleep health [100]. While innovative, this study relied on self-reported dietary and sleep data and was drawn from a health-conscious user base, limiting generalisability to perimenopausal women [99].

Protein and choline intake may improve sleep quality, with inadequate intakes linked to poorer sleep and increased daytime sleepiness [101]. Choline supports rapid eye movement (REM) sleep by facilitating the production of acetylcholine, a neurotransmitter critical to the regulation of the sleep–wake cycle [75]. Notably, three studies have highlighted the negative impact of insufficient protein and choline on sleep outcomes [70,99,101]. In one observational study using National Health and Nutrition Examination Survey (NHANES) 2007–2008 data, pre- and perimenopausal women (*n* = 1116) who consumed lower amounts of protein and choline were significantly more likely to report very short sleep durations (<5 h) [70]. An increase of 20 g·day^−1^ in total protein intake was associated with an 83% lower risk of very short sleep, while an increase of about 100 mg·day^−1^ of total choline intake was associated with a 74% lower risk of very short sleep [70]. While these findings are compelling, the correlational design, reliance on self-report measures and use of a single 24 h diet recall and combining of pre- and perimenopausal women limit the specificity of the conclusions. There is additional evidence from a 2017 exploratory case–control study comparing plasma choline levels in 36 adults (mean age 42.4 years; 50% women) with and without excessive daytime sleepiness [101]. Participants in the “sleepy” group (Epworth Sleepiness Scale; ESS ≥ 10) had significantly lower plasma choline levels than those without sleepiness [101]. These findings suggest that low choline levels may be associated with sleep-related symptoms, although the small sample size and lack of hormonal stratification limit interpretation. From a practical perspective, the magnitude of change associated with better sleep outcomes in the NHANES study, roughly 20 g more protein and 100 mg more choline per day, could feasibly be achieved through one to three additional eggs or equivalent protein sources daily, making this a realistic dietary strategy for midlife women.

Beyond sleep, dietary protein also plays a crucial role in appetite regulation and the preservation of healthy body composition, factors of growing importance during perimenopause due to hormonal changes, reduced energy expenditure, and increased risk of central adiposity [102]. Conceptual and empirical work during the menopause transition supports a “protein-leverage mechanism”: when the proportion of dietary protein is insufficient, a biologically driven appetite for protein leads to compensatory intake of carbohydrate and fat, promoting weight gain and loss of lean mass [103]. With 12.6 g of high-quality protein per two units of eggs (Table 2), they represent a simple and convenient way to increase the protein density of meals without substantially increasing total energy intake (~140 kcal), supporting the protein-leverage effect whereby higher protein proportion may promote satiety and reduce subsequent energy intake [104]. When incorporated as part of a balanced diet providing ≥1.2·g·kg^−1^·day^−1^ of protein, eggs can help improve daily protein distribution and complement physical activity strategies aimed at preserving lean mass and attenuating fat gain during the menopausal transition [105]. A 2016 meta-analysis of five isocaloric preload studies (including 21 women aged >40 years) demonstrated that higher-protein preloads (i.e., ~20–50 g protein, or ~25–30% of total energy) significantly increased fullness compared with lower-protein preloads (0–10 g protein; ~5% energy), independent of total energy intake [106]. A 12-day randomised crossover trial in 79 adults (mean age: 34.0 ± 17.6 years, 39 women) also showed that diets with higher protein contribution (i.e., 30% of total energy) reduced energy intake without increasing hunger [107]. While short and largely reliant on subjective appetite ratings, these findings align with protein-leverage predictions [103] and support a pragmatic focus on protein quality and the contribution of energy in midlife women.

Taken together, protein and choline, both present in eggs, may contribute to sleep regulation, reduce daytime sleepiness, enhance satiety, and support favourable body-composition changes (i.e., preserving lean mass while limiting fat accumulation) during perimenopause. However, further research is needed to clarify optimal intake levels, timing, and food-based strategies in women during this life stage.

### 5.2. Tryptophan, Melatonin and Vitamin D

Eggs provide approximately 160 mg of tryptophan, 3.08 ng of melatonin, and 8.2 µg of vitamin D per two eggs (Table 2), all of which are implicated in circadian rhythm regulation and neurotransmitter pathways supporting serotonin and melatonin synthesis [73]. Although eggs contain small amounts of melatonin, tryptophan is likely the primary driver of any potential sleep-supporting effects, due to its well-established role as a precursor to serotonin and melatonin [26,76]. This role of tryptophan in the regulation of serotonin/melatonin synthesis means that, in Australia; the Therapeutic Goods Administration regulates preparations containing isolated tryptophan at a dose greater than 100 mg·day^−1^ as prescription medicines [108]. Therefore, to supplement the diet with a bioactive dose of tryptophan without a prescription, one is restricted to whole food sources. Several studies have investigated the effects of these individual nutrients on sleep quality and duration, although research specifically targeting perimenopausal women remains scarce. A 2024 cross-sectional study assessed the relationship between tryptophan intake and sleep parameters in 11,485 Spanish university students (72.7% women, mean age: 20 years) [109]. Tryptophan intake was estimated using a validated food intake matrix (a tool that links reported foods with nutrient content to evaluate dietary intake) and categorised into quartiles, while sleep parameters, including duration (<7 h as short sleep), efficiency, insomnia symptoms, and Athens Insomnia Scale (AIS, range: 0–24) scores were self-reported [109]. Women in the lowest quartile of tryptophan intake (<526 mg·day^−1^) had a significantly higher risk of short sleep duration (Odd ratio (OR): 1.19; 95% CI: 1.05–1.34) and elevated AIS scores (OR: 1.47; 95% CI: 1.10–2.05) [109]. These findings suggest that tryptophan intake may modestly influence sleep quality and duration in university students. Supporting these findings, a 2022 meta-analysis of 21 studies (*n* = 522 participants) found that tryptophan supplementation significantly reduced wake time after sleep onset by an average of 81 min per gram (*p* = 0.017) [110]. Notably, doses >1 g·day^−1^ were more effective than lower doses (56.55 vs. 28.91 min reduction in wake time, *p* = 0.001). However, these studies involved isolated tryptophan supplementation rather than whole foods, limiting applicability to dietary sources like eggs, which also contain other branched-chain amino acids which compete with tryptophan for entry into the brain [110]. Additionally, the Spanish university study cohort was young, healthy, and self-reporting, with limited adjustments for important sleep confounders (e.g., alcohol use, physical activity), reducing generalisability to perimenopausal populations. Beyond sleep outcomes, evidence linking tryptophan to body composition in women is limited; in a randomised crossover trial of healthy young women, L-tryptophan up to 5 g·day^−1^ did not change food intake or body mass [111]. Taken together, these limitations highlight the need for well-controlled food-based intervention trials assessing tryptophan-rich foods, such as eggs, in the regulation of perimenopausal symptoms such as sleep disturbances.

Direct evidence on melatonin from eggs is limited; however, food-based interventions provide useful proof of concept that even sub-pharmacological doses of bioactive compounds may influence sleep. Studies using Montmorency tart cherry juice, another food naturally containing melatonin and tryptophan, have shown modest improvements in sleep, including increases in total sleep time (+84 min, *p* = 0.02) and subjective sleep efficiency (~10%, *p* = 0.03) after twice-daily intake, alongside higher overnight urinary melatonin concentrations and longer sleep duration (~22 min, *p* < 0.05) following 7 days of supplementation [87]. Although these changes occurred at melatonin (~0.135 µg·100 g^−1^) and tryptophan (~9 mg·100 g^−1^) levels far below typical supplemental doses (0.5–5 mg and 1.2–2.4 g, respectively) [112,113], the findings demonstrate that whole-food matrices containing small quantities of these compounds can modulate physiological outcomes such as sleep. This suggests that potential effects of eggs, whose melatonin content is similarly low, may arise not from pharmacological dosing but from synergistic interactions among multiple nutrients or indirect modulation of tryptophan-melatonin metabolism.

When considering body composition outcomes, a recent systematic review of randomised trials found no significant effects of tart cherry juice on body mass, body mass index, fat mass, fat-free mass, waist circumference, or per cent body fat in adults with heterogeneous age ranges and no perimenopause-specific cohorts [114]. Given the different food matrix and small sample sizes (*n* = 8–34), these data primarily serve as proof of concept rather than direct evidence applicable to eggs, reinforcing the need for egg-specific trials.

Vitamin D, widely recognised for its immune health benefits [115], also appears to play a role in sleep regulation. Two eggs provide approximately 82% of the recommended daily intake of vitamin D, underscoring their potential contribution to sleep health [28,116]. Several cross-sectional studies have reported shorter sleep duration in individuals with lower circulating vitamin D levels [98,117]. More recently, a 2023 study in Iranian adults (*n* = 535; 46% women; mean age 42.57 years) examined dietary nutrient patterns, serum 25-hydroxyvitamin D concentrations, and sleep duration and quality assessed via the PSQI [118]. Participants with inadequate vitamin D status (<20 ng·mL^−1^ serum 25-hydroxyvitamin D) and low adherence to a high-vegetable dietary pattern were more than three times as likely to report short sleep (OR: 3.42; 95% CI: 1.42–6.64). Supporting these findings, a meta-analysis of nine studies (*n* = 9397; 36% women) reported that vitamin D deficiency (defined between 10 and 30 ng·mL^−1^) was associated with a significantly increased risk of sleep disorders (OR: 1.50; 95% CI: 1.31–1.72), including poor sleep quality (OR: 1.59; 95% CI: 1.23–2.05), short sleep duration (OR: 1.74; 95% CI: 1.30–2.32), and excessive sleepiness (OR: 1.36; 95% CI: 1.12–1.65) [119]. While these findings are mostly observational, they suggest that ensuring adequate vitamin D intake, including from food sources such as eggs, may be an accessible strategy to support sleep health.

Across the menopausal transition, shifts in body composition also coincide with a higher prevalence of low vitamin D, and higher adiposity tends to lower circulating vitamin D [120]. In peri- and postmenopausal women, a 30-month double-blind RCT showed that daily calcium (500 mg) plus vitamin D (200 IU) supplementation preserved total-body bone mineral density and content compared with placebo (placebo lost ~0.4% bone mineral density per year), reinforcing the value of maintaining calcium and vitamin D status, alongside practical food sources such as eggs, to support musculoskeletal health [121]. Although vitamin D can be synthesised endogenously through ultraviolet B (UVB) radiation from sunlight exposure, this process often becomes insufficient due to low sun exposure, seasonal variation, and the use of protective clothing [122]. Furthermore, hepatic hydroxylation of vitamin D precursors decreases with age, reducing the efficiency of vitamin D activation [44]. Notably, a recent trial in Australian adults (*n* = 51; 75% women, average age 30 years) demonstrated that regular egg consumption (7 eggs per week) over 12 weeks during winter helped maintain serum 25-hydroxyvitamin D concentrations, suggesting that eggs may contribute meaningfully to vitamin D status when UVB exposure is limited [19]. Therefore, ensuring adequate dietary intake from sources such as eggs, becomes increasingly important to maintain optimal vitamin D status in midlife women. If perimenopausal women engage in weight-management efforts, maintaining sufficient vitamin D can help preserve calcium absorption under energy restriction and may support muscle function [120].

### 5.3. Antioxidants

Perimenopause brings fluctuating oestrogen levels that can elevate oxidative stress, contributing to inflammation, disrupted sleep, and shifting body fat distribution [123,124]. Eggs contain several natural antioxidant compounds, like egg-yolk carotenoids, lutein and zeaxanthin, egg-white proteins including ovalbumin, ovotransferrin, lysozyme, and yolk phosvitin, that support cellular protection in midlife women [125]. Notably, lutein and zeaxanthin are highly absorbable from enriched eggs and linked to reduced oxidative stress in tissues, including the skin and retina [126], which may help maintain vascular and metabolic health during hormonal transitions. Egg-white ovotransferrin, and especially its digestion-derived peptides, exhibit enhanced antioxidant and metal-chelating properties, which support protection against oxidative damage in vitro [127]. Emerging evidence further supports a role for antioxidants in sleep regulation. Analysis of NHANES data found that higher dietary antioxidant intake, particularly selenium, vitamin C, and vitamin E, was associated with up to a 14% lower risk of sleep disorders [128]. Similarly, a recent review reported that natural antioxidants, including carotenoids, flavonoids, and vitamins C and E, reduce sleep onset latency, duration, and efficiency of approximately 8–15% by reducing oxidative stress and inflammation, with specific benefits observed in postmenopausal women [129]. Taken together, eggs provide several antioxidant compounds that may help counteract oxidative stress. Although the antioxidants examined in sleep-related studies (e.g., vitamins C and E) differ from those found in eggs, the shared oxidative pathways suggest a plausible supportive role for egg-derived antioxidants [125].

Collectively, the evidence outlined above illustrates how hormonal fluctuations during perimenopause interact with metabolic regulation, appetite, and sleep behaviour in a way that makes diet and nutrient timing increasingly relevant. Declining oestrogen and progesterone contribute to circadian instability, altered thermoregulation, and shifts in appetite hormones, while sleep disruption amplifies changes in energy balance and food choices. Within this context, whole-food sources that provide high-quality protein and sleep-relevant micronutrients, such as eggs, may help support overnight recovery, appetite control, and metabolic stability, particularly when incorporated later in the day. While these interrelationships remain a working hypothesis rather than an established causal pathway, considering hormonal changes, diet, and sleep as interconnected offers a coherent lens through which to interpret subsequent evidence on perimenopausal symptoms and cardiometabolic health.

## 6. Potential Roles of Egg Nutrients in Managing Perimenopausal Symptoms

Although eggs have been explored for their nutritional benefits, their role in managing broader perimenopausal symptoms (Table 1) beyond sleep remains under-researched and inconclusive. A recent survey of 52,347 Chinese women aged 35–60 years found that higher daily egg consumption, assessed via a food frequency questionnaire, was initially associated with a delayed onset of natural menopause [130]. However, this relationship lost significance after adjusting for confounding variables (i.e., age, body mass index, menopausal status, marital status, smoking and alcohol use, education level, physical activity, sleep quality, fruit and vegetable intake, sugar-sweetened beverage consumption, and disease history), suggesting that the observed association may have been influenced by other dietary and/or lifestyle factors [130]. Similarly, no significant correlations were observed between egg intake and the severity of menopausal symptoms, but the reliance on self-reported dietary intake introduced subjectivity, limiting the strength of any conclusions [130]. Due to the reliance on subjective, self-reported data, no definitive conclusions can be drawn about the impact of egg consumption on menopause-related outcomes from these studies, highlighting the need for more rigorous, high-quality research.

While research specifically linking egg intake to perimenopausal symptoms is lacking, there is broader evidence that dietary patterns can influence the experience of menopause. A systematic review of 19 studies (*n* = 288 women) reported that higher consumption of ultra-processed foods, sugar-sweetened beverages, and processed meats was associated with more severe vasomotor, somatic, sexual, and cognitive symptoms in postmenopausal women [131]. In contrast, greater vegetable intake was associated with improved sleep, mood, and overall quality of life [131]. In perimenopausal women, one clinical trial demonstrated that a combined intervention including the Dietary Approaches to Stop Hypertension (DASH) diet, health education, and resistance training led to improvements in a range of menopausal symptoms such as hot flushes, sweating, irritability, depression, fatigue, joint pain, muscle pain, palpitations and sexual disorders after three months [43]. However, eggs were not specifically included in the described dietary patterns, reflecting a gap in the literature on their potential utility in this context.

Eggs may also support mood regulation during perimenopause due to their vitamin D content, a nutrient that has been linked to mental health and lower depression risk [132]. In a large prospective analysis from the Women’s Health Initiative involving 81,189 women aged 50–79 years (some likely perimenopausal at baseline), those who consumed 400 IU of vitamin D per day (~10 µg; or roughly 67% of the recommended dietary allowance for adults) primarily from food sources, had a 20% lower risk of developing depressive symptoms over three years compared with those consuming only 100 IU [132]. For context, two eggs will provide ~5 µg of vitamin D equivalent [19] or ~200 IU [133]. Depressive symptoms were assessed using the validated Burnam depression scale alongside assessments of current antidepressant medication use. While the specific food sources of vitamin D were not detailed, these findings suggest that regular food-based vitamin D, such as that found in eggs, may offer a protective effect against mood-related symptoms during the menopausal transition. Beyond sleep, regular egg intake may also positively influence other perimenopausal symptoms such as mood fluctuations, fatigue, and hot flushes [55,73,134]. Given their broad nutritional profile, including protein, vitamin D, choline, and essential fatty acids, eggs represent a potentially underutilised dietary tool for supporting health and quality of life in midlife women.

## 7. Cardiometabolic Considerations and Implications for Practice

While eggs provide high-quality protein and essential nutrients, concerns about their cholesterol content remain, especially for women approaching or undergoing menopause, due to the known increased cardiovascular risk during this life stage [135]. Because cardiometabolic health strongly influences sleep quality and overall well-being during midlife, understanding these interactions is particularly relevant to the present review. A key source of controversy is that foods high in cholesterol are often also high in saturated fat [94], which independently increases low-density lipoprotein cholesterol (LDL; “bad” cholesterol) and cardiovascular disease risk [136]. This makes it difficult to isolate the effects of dietary cholesterol from those of saturated fat. Eggs, however, are distinct in being high in cholesterol but relatively low in saturated fat; approximately 244 mg of cholesterol and only 1.2 g of saturated fat per large egg (50 g) [95]. Because eggs are consumed within broader dietary patterns, associated foods and lifestyle habits strongly influence outcomes. In Western diets, eggs are often eaten with processed meats high in saturated fat (e.g., bacon or sausage) [96], whereas in Asian populations, egg intake is more frequently associated with higher socioeconomic status, greater physical activity, and healthier dietary habits [97,137].

In a large prospective study of U.S. postmenopausal women from the Women’s Health Initiative (*n* = 84,949; mean age 63 years), higher dietary cholesterol intake was associated with a modestly elevated risk of incident cardiovascular disease and all-cause mortality [138]. Specifically, greater cholesterol intake was linked with higher risks of ischemic heart disease, ischemic stroke, and cardiovascular disease mortality, while an inverse association was observed for haemorrhagic stroke, and no association was found with deaths from cancer, dementia, or respiratory diseases. The patterns of association for egg consumption were comparable to those of dietary cholesterol, with higher egg intake (≥1 egg·day^−1^ vs. <1 egg·week^−1^) accounting for roughly 60% of total dietary cholesterol in this cohort [138]. Mechanistically, these relationships may relate to changes in circulating lipid profiles, particularly higher LDL cholesterol concentrations, which are a well-established contributor to ischemic cardiovascular disease, while lower LDL cholesterol concentrations have been linked to greater haemorrhagic stroke risk [138]. These findings suggest that, for some women, particularly those with existing risk factors, moderation in egg intake may be prudent. Importantly, individual variability in lipid responses is well described, with a subset of individuals acting as “hyper-responders” who show greater increases in circulating cholesterol following higher dietary cholesterol intake. However, the broader evidence remains mixed. A global study involving over 177,000 participants found no significant link between moderate egg intake (up to 7 eggs·week^−1^) and cardiovascular disease or mortality across diverse populations [139]. Moreover, egg nutrients such as phospholipids and unsaturated fats may confer cardiovascular benefits that offset cholesterol concerns [140]. Notably, higher egg consumption may increase risk in individuals with diabetes [141,142,143], although these associations are often attenuated after adjusting for background diet [144].

Current dietary guidelines, including those from the American Heart Association (2019) and the U.S. Dietary Guidelines Advisory Committee (2020–2025), indicate that moderate egg consumption, approximately one egg per day, is compatible with healthy lipid profiles and cardiovascular outcomes [145,146]. Contemporary evidence similarly shows that, for most healthy adults, moderate egg consumption does not adversely affect serum LDL cholesterol or cardiovascular risk, likely due to concurrent rises in high-density lipoprotein (HDL; “good” cholesterol) and the high nutrient density of eggs [147,148]. However, there is considerable individual variability. This variability, together with inconsistent findings in cohort studies across populations, underscores the need to consider baseline cardiovascular risk and dietary context when interpreting egg-related outcomes. Consistent with these findings, the Australian Heart Foundation’s 2023 position statement [149] concludes that, for healthy individuals, eggs can be included regularly (up to seven per week) within a heart-healthy eating pattern, emphasising that overall diet quality is more important for cardiovascular risk than limiting single foods or nutrients. Taken together, dietary context and individual risk factors should guide intake, rather than applying absolute restrictions.

Although research specifically in perimenopausal women remains limited, egg-based diets are generally well-tolerated and widely accepted [60,90,91,92,150]. For instance, a 2021 randomised controlled trial, in adults with type-2 diabetes (*n* = 15, 10 women, mean age: 64 years) compared three bedtime snack conditions across 3-day periods: two cooked eggs, two fruit-flavoured Greek yoghurts (150 kcal), or no snack. Results showed that two eggs in the evening, relative to a high-carbohydrate snack, improved fasting glucose and insulin sensitivity and was well tolerated, although the study did not specifically include perimenopausal participants [151]. Recent data from a large cohort of adults with sleep disorders further suggest that consuming eggs and milk in the evening may be associated with a 28% lower risk of cardiovascular mortality, highlighting the potential importance of meal timing during midlife, particularly for women experiencing sleep disturbances [60]. However, the same study noted increased all-cause and cancer mortality when egg intake exceeded ~1.5 eggs·day^−1^ or dietary cholesterol surpassed 250 mg·day^−1^ [60]. However, as this finding is derived from NHANES data, it likely reflects broader dietary and lifestyle patterns, given that eggs are commonly consumed within mixed meals, rather than a direct effect of egg intake alone. Taken together, the overall evidence supports a favourable risk–benefit profile for eggs when consumed in moderation as part of a balanced diet. While excessive intakes may increase serum cholesterol in susceptible individuals, eggs deliver high-quality protein, vitamin D, choline, carotenoids, and unsaturated fats that collectively support cardiometabolic, musculoskeletal, and cognitive health. Recent analyses indicate no association between moderate egg intake and cardiovascular disease or mortality, and leading authorities, including the Australian Heart Foundation [149], emphasise that overall dietary quality, rather than the inclusion of specific foods such as eggs, is the key determinant of cardiovascular risk [149,152]. In this context, the nutritional benefits of eggs may outweigh potential risks for most healthy individuals, particularly when prepared with minimal added fats and consumed within a varied, whole-food dietary pattern.

With growing interest in protein timing and targeted nutrition during the perimenopausal transition, further research may help position eggs as a practical and beneficial option for evening meals in this population, provided intake is aligned with individual health status and consumed as part of a balanced dietary pattern.

## 8. Limitations of Current Literature

The existing evidence examining egg intake and sleep remains scarce and methodologically limited. Few studies have specifically targeted peri- or postmenopausal women, restricting the ability to generalise findings to midlife women undergoing the menopausal transition. Most studies rely on self-reported dietary intake and subjective sleep measures, both prone to recall and reporting bias. The predominance of cross-sectional or observational designs further constrains causal inference. Additionally, inconsistencies in reporting the quantity, frequency, and timing of egg consumption make it difficult to identify potential dose–response or timing effects. Cultural differences in dietary patterns and food availability may also reduce the applicability of findings to Western perimenopausal populations, as most existing studies have been conducted in East Asian cohorts, particularly in China, Japan, and Korea. Finally, as this review is narrative in design and not preregistered or synthesised using quantitative methods, it is susceptible to selection bias and cannot provide pooled estimates or formal bias diagnostics. These constraints should be considered when interpreting the conclusions, which reflect integrative interpretation rather than systematic evidence aggregation.

## 9. Future Directions and Practical Recommendations

Despite the aforementioned limitations, several key points can help guide practice. For dietitians, nutritionists, and general practitioners working with perimenopausal women, current evidence does not yet support specific dietary recommendations; however, moderate egg consumption can be included as part of a nutrient-dense dietary pattern that aligns with national dietary guidelines and may support overall health and sleep. Importantly, eggs should not be viewed as a stand-alone sleep intervention, and findings to date remain preliminary. Briefly, practitioners should also consider potential health risks for specific populations, such as cholesterol concerns in individuals with hypercholesterolemia or other cardiometabolic conditions, and tailor recommendations accordingly.

Future studies should specifically recruit peri- and postmenopausal women to address the current gap in age- and sex-specific evidence. Randomised controlled trials using objective sleep measures (e.g., actigraphy, polysomnography) are needed to clarify whether eggs influence sleep outcomes and to what extent, with primary outcomes such as sleep efficiency, sleep onset latency, wake after sleep onset, and sleep architecture. Clearer reporting of egg quantity, preparation method, and timing of intake will help establish practical recommendations. Research should also examine whether evening consumption, compared with morning intake, has distinct effects on sleep physiology, ideally over multi-week interventions (e.g., 4–12 weeks or more) with appropriate comparator groups such as isocaloric non-egg protein foods or habitual diet controls. Finally, exploring interactions between egg consumption, other dietary patterns, and hormonal status could provide a more nuanced understanding of how whole foods can support sleep health during midlife.

## 10. Conclusions

Current research on evening egg intake and sleep outcomes remains limited, particularly in perimenopausal women, a group disproportionately affected by sleep disturbances. This highlights both the need for controlled research in this population and the broader gap in the understanding of nutrition-based strategies to support sleep. Given the physiological and hormonal changes during perimenopause, dietary interventions hold significant potential to improve sleep quality and alleviate symptoms, ultimately enhancing quality of life during midlife and beyond. The nutrient composition of eggs suggests they could improve sleep regulation, duration, and mood during this transitional period. Investigating the effects of evening egg consumption through well-designed, food-based studies could offer valuable insights and contribute to practical strategies for healthy ageing and reducing perimenopausal symptoms.

## Figures and Tables

**Table 1:** Symptoms experienced during perimenopause.

Category	Common Symptoms
Neurological	Headaches.
Energy and sleep	Physical exhaustion, mental exhaustion, difficulty initiating or maintaining sleep (e.g., latency, duration, fragmentation).
Mood and cognitive function	Mood swings, low mood, anxiety, irritability, difficulty concentrating, brain fog and memory lapses.
Hair, skin, and nails	Hair thinning/loss, brittle nails, breakouts, pruritus/skin irritation, dry skin, facial wrinkles, reduced skin firmness, hyperpigmentation (dark spots).
Vasomotor symptoms	Hot flushes.
Gastrointestinal and genitourinary	Bloating, reflux/heartburn, abdominal discomfort, constipation, diarrhoea and urinary urgency, frequency or incontinence.
Musculoskeletal	Joint stiffness, chronic back pain, muscle pain, and generalised aches.
Sexual and Reproductive Health	Reduced libido, vaginal dryness, dyspareunia (painful intercourse).
Body composition and musculoskeletal health	Weight gain, increased visceral adiposity, and bone and muscle loss.

Adapted from: [1,42,43,44].

## Supplementary Materials

The following are available online at https://www.mdpi.com/article/10.3390/nu17243837/s1, Table S1: Search Strategy, inclusion, and exclusion criteria.
