# Supplementary material for: Eggs as a Nutrient-Rich Food with Potential Relevance to Sleep Metabolic Health, and Well-Being During the Menopausal Transition: A Narrative Review
Source: Nutrients. 2025 Dec 8;17(24):3837. doi: 10.3390/nu17243837 (PMC12735762; doi:10.3390/nu17243837)
Supplement: Supplementary file 1 [file nutrients-17-03837-s001.zip › nutrients-4004440-supplementary.pdf]

Supplementary Table S1. Search Strategy, inclusion, and exclusion criteria

Search Engine: PubMed, Scopus, Medline Complete, Embase

**PICOS** Criteria	**Search Strategy**	**Inclusion Criteria**	**Exclusion Criteria**
Population	"female" OR "wom?n" OR "perimenopause*" OR "menopaus*" OR "Menopause transition"	- female healthy adults (≥40 years)<br>- naturally menstruating, hormonal contraceptive users and hormonal replacement therapies<br>- with current perimenopause symptoms and/or menstrual cycle disturbances<br>- or menopausal	- involved animal models, and<br>- were published in a language other than English.
AND			
Intervention	"egg*" OR "egg* consumption" OR "dietary egg"		
AND			
Comparator		- with comparisons to suitable controls (e.g., control condition or pre- and post-intervention assessment)	
AND			
Outcomes		- any sleep or health outcome associated with consuming eggs during peri/menopause,	
AND			
Study Design		- Original research: e.g., Randomised controlled trial (RCT) Clinical trial, crossover study, observational study	- were not original research, e.g., abstracts, thesis, commentaries

Manuscript ID: nutrients-4004440
